# Supplementary material for: Disease Progression and Serological Assay Performance in Heritage Breed Pigs following Brucella suis Experimental Challenge as a Model for Naturally Infected Feral Swine
Source: Pathogens. 2023 Apr 24;12(5):638. doi: 10.3390/pathogens12050638 (PMC10221608; doi:10.3390/pathogens12050638)
Supplement: Supplementary file 1 [file pathogens-12-00638-s001.zip › pathogens-2306262-SI.pdf]

pathogens

MDPI

Table S1. Evaluation of difference among sex in the Ossabaw Island Hog tissues infected by *B. suis* and the intensity of infection using Fisher's exact probability test of binomial proportions (number of tissues infected) and the mean bacterial count and number of tissues infected using Fisher's exact Poisson test. Results that are significant at the 0.05 level are noted in bold

Tissue	Tissues Infected					Bacterial Count				
	**Male**	***n***	**Female**	***n***	***P*–Value**	**Male**	***n***	**Female**	***n***	***P*–Value**
Mandibular lymph node	9	11	7	12	0.221	113.8	11	70.5	12	$\mathbf{9.20 \times 10^{-28}}$
Retropharyngeal lymph node	7	11	9	12	0.445	103.2	11	38.8	12	$\mathbf{4.37 \times 10^{-78}}$
Parotid lymph node	9	11	9	12	0.500	105.6	11	7.9	12	$\mathbf{6.34 \times 10^{-255}}$
Mediastinal lymph node	8	11	6	12	0.246	1.9	11	87.2	12	$\mathbf{3.43 \times 10^{-259}}$
Mesenteric lymph node	4	11	5	12	0.500	38.9	11	18.5	12	$\mathbf{1.63 \times 10^{-20}}$
Mammary/Inguinal lymph node	7	11	7	12	0.500	27.6	11	50.2	12	$\mathbf{1.81 \times 10^{-18}}$
Spleen	6	11	7	12	0.500	61.0	11	88.2	12	$\mathbf{2.70 \times 10^{-14}}$
Liver	7	11	5	12	0.262	8.4	11	4.2	12	$\mathbf{3.46 \times 10^{-05}}$
Lung	7	11	5	12	0.262	10.4	11	4.8	12	$\mathbf{5.17 \times 10^{-07}}$
Peripheral blood mononuclear cell	4	11	5	12	0.500	2.3	11	6.0	12	$\mathbf{7.07 \times 10^{-06}}$
All Tissues	6.2	11	5.8	12	0.665	473.1	11	377.2	12	$\mathbf{3.34 \times 10^{-29}}$

Table S2. Evaluation of difference among age in the Ossabaw Island Hog tissues infected by *B. suis* and the intensity of infection using Fisher's exact probability test of binomial proportions (number of tissues infected) and the mean bacterial count and number of tissues infected using Fisher's exact Poisson test. Results that are significant at the 0.05 level are noted in bold.

Tissue	Tissues Infected					Bacterial Count				
	**Adult**	***n***	**Subadult**	***n***	***P*–Value**	**Adult**	***n***	**Subadult**	***n***	***P*–Value**
Mandibular lymph node	12	18	4	5	0.510	83.8	18	117.8	5	1
Retropharyngeal lymph node	13	18	3	5	0.500	41.3	18	171.4	5	$\mathbf{2.82 \times 10^{-169}}$
Parotid lymph node	14	18	4	5	0.500	22.2	18	171.4	5	$\mathbf{2.26 \times 10^{-271}}$
Mediastinal lymph node	11	18	3	5	0.500	58.9	18	1.2	5	$\mathbf{2.45 \times 10^{-102}}$
Mesenteric lymph node	8	18	1	5	0.318	32.1	18	14.4	5	$\mathbf{9.39 \times 10^{-13}}$
Mammary/Inguinal lymph node	10	18	4	5	0.682	39.1	18	40.8	5	0.724
Endometrium	3	11	0	1	0.500	0.4	11	0	1	0.706
Vaginal swab	2	11	0	1	0.500	0.6	11	0	1	0.544
Spleen	11	18	2	5	0.370	66	18	108.2	5	1
Liver	9	18	3	5	0.500	5.7	18	7.8	5	0.957
Lung	9	18	3	5	0.500	7.3	18	7.8	5	0.672
Peripheral blood mononuclear cell	8	18	1	5	0.318	4.6	18	3	5	0.0807
All Tissues	6.1	18	5.6	5	0.385	361.7	18	643.8	5	1

Table S3. Evaluation of difference among strain in the Ossabaw Island Hog tissues infected by *B. suis* and the intensity of infection using Fisher's exact probability test of binomial proportions (number of tissues infected) and the mean bacterial count and number of tissues infected using Fisher's exact Poisson test. Results that are significant at the 0.05 level are noted in bold.

**Tissue**	**Tissues Infected**					**Bacterial Count**				
	**1330**	***n***	**294**	***n***	***P*–Value**	**1330**	***n***	**294**	***n***	***P*–Value**
Mandibular lymph node	8	12	8	11	0.500	133.8	12	44.7	11	**2.44×10^{-117}**
Retropharyngeal lymph node	8	12	8	11	0.500	96.5	12	40.3	11	**3.83×10^{-61}**
Parotid lymph node	9	12	9	11	0.500	74	12	33.5	11	**9.76×10^{-41}**
Mediastinal lymph node	8	12	6	11	0.434	4.4	12	92.2	11	**3.30×10^{-250}**
Mesenteric lymph node	4	12	5	11	0.434	6.7	12	51.8	11	**6.92×10^{-102}**
Mammary/Inguinal lymph node	8	12	6	11	0.434	23.6	12	56.7	11	**2.17×10^{-37}**
Endometrium	3	7	0	5	0.155	0.6	7	0	5	0.116
Vaginal swab	0	7	2	5	0.147	0	7	1.4	5	**0.00218**
Spleen	8	12	5	11	0.273	49.9	12	102.7	11	**7.21×10^{-49}**
Liver	8	12	4	11	0.150	6.3	12	6	11	0.407
Lung	8	12	4	11	0.150	5.8	12	9.2	11	**0.00207**
Peripheral blood mononuclear cell	5	12	4	11	0.500	6.3	12	1.9	11	**8.87×10^{-08}**
All Tissues	6.4	12	5.5	11	0.222	407.8	12	439.7	11	1

Table S4. Diagnostic assay performance using a single assay.

Assay	Category	Sensitivity	Specificity	Positive Predictive Value	Negative Predictive Value	Diagnostic Odds Ratio
Buffered Acidified Plate Antigen	All Animals	0.980 (0.909–0.984)	0.833 (0.636–0.969)	0.891 (0.754–0.981)	0.967 (0.857–0.977)	8.2 (3.1–51.0)
Card Test (8%)	All Animals	0.917 (0.784–0.982)	0.935 (0.786–0.979)	0.953 (0.833–0.983)	0.891 (0.719–0.977)	20.0 (5.0–58.0)
Complement Fixation (Cold)	All Animals	0.970 (0.941–0.980)	0.730 (0.511–0.900)	0.836 (0.688–0.945)	0.964 (0.941–0.975)	5.1 (2.2–17.7)
Fluorescent Polarization Assay	All Animals	0.564 (0.429–0.692)	0.701 (0.548–0.835)	0.723 (0.578–0.850)	0.539 (0.402–0.674)	2.6 (1.4–5.5)
Buffered Acidified Plate Antigen	Adult	0.976 (0.894–0.981)	0.885 (0.636–1.000)	0.946 (0.810–1.000)	0.944 (0.769–0.967)	17.0 (4.4–Inf)
Card Test (8%)	Adult	0.919 (0.767–0.979)	0.957 (0.923–1.000)	0.975 (0.966–1.000)	0.864 (0.625–0.967)	39.0 (28.0–Inf)
Complement Fixation (Cold)	Adult	0.963 (0.929–0.974)	0.840 (0.577–1.000)	0.925 (0.784–1.000)	0.947 (0.900–0.966)	12.7 (3.6–Inf)
Fluorescent Polarization Assay	Adult	0.578 (0.430–0.722)	0.745 (0.550–0.906)	0.817 (0.661–0.934)	0.472 (0.307–0.635)	4.5 (2.0–14.2)
Buffered Acidified Plate Antigen	Subadult	0.900 (0.750–0.941)	0.778 (0.444–1.000)	0.727 (0.333–1.000)	0.923 (0.857–0.947)	2.7 (0.5–Inf)
Card Test (8%)	Subadult	0.875 (0.500–0.938)	0.846 (0.526–1.000)	0.769 (0.333–1.000)	0.923 (0.667–0.950)	3.3 (0.5–Inf)
Complement Fixation (Cold)	Subadult	0.900 (0.800–0.938)	0.583 (0.267–0.905)	0.579 (0.263–0.909)	0.900 (0.800–0.941)	1.4 (0.3–10.0)
Fluorescent Polarization Assay	Subadult	0.500 (0.190–0.812)	0.654 (0.406–0.857)	0.450 (0.167–0.750)	0.694 (0.441–0.897)	0.8 (0.2–3.0)
Buffered Acidified Plate Antigen	Female	0.964 (0.848–0.973)	0.846 (0.526–1.000)	0.920 (0.731–1.000)	0.923 (0.692–0.957)	11.5 (2.8–Inf)
Card Test (8%)	Female	0.879 (0.667–0.970)	0.941 (0.889–1.000)	0.962 (0.944–1.000)	0.824 (0.538–0.957)	25.0 (16.0–Inf)
Complement Fixation (Cold)	Female	0.947 (0.875–1.000)	0.720 (0.375–1.000)	0.867 (0.667–1.000)	0.917 (0.800–0.952)	6.6 (2.0–Inf)
Fluorescent Polarization Assay	Female	0.614 (0.435–0.780)	0.651 (0.406–0.865)	0.766 (0.579–0.911)	0.475 (0.279–0.686)	3.3 (1.4–10.2)
Buffered Acidified Plate Antigen	Male	0.957 (0.933–0.969)	0.840 (0.571–1.000)	0.870 (0.633–1.000)	0.947 (0.900–0.963)	6.7 (1.8–Inf)
Card Test (8%)	Male	0.952 (0.800–0.968)	0.889 (0.625–1.000)	0.900 (0.667–1.000)	0.944 (0.773–0.964)	9.3 (2.0–Inf)
Complement Fixation (Cold)	Male	0.929 (0.889–0.952)	0.750 (0.450–0.955)	0.806 (0.562–0.968)	0.941 (0.889–0.960)	4.2 (1.3–30.0)
Fluorescent Polarization Assay	Male	0.500 (0.297–0.700)	0.744 (0.543–0.902)	0.667 (0.436–0.872)	0.586 (0.400–0.763)	2.0 (0.8–6.8)

Table S5. Diagnostic assay performance when two assays are interpreted in parallel.

Assay	Assay	Sensitivity	Specificity	Positive Predictive Value	Negative Predictive Value	Diagnostic Odds Ratio	Area Under Curve
Buffered Acidified Plate Antigen	Complement Fixation (Cold)	0.980 (0.914–0.984)	0.833 (0.635–0.969)	0.891 (0.754–0.981)	0.967 (0.857–0.977)	8.3 (3.1–50.0)	0.981 (0.884–0.999)
Card Test (8%)	Buffered Acidified Plate Antigen	0.898 (0.753–0.981)	0.936 (0.780–0.978)	0.952 (0.832–0.983)	0.869 (0.692–0.975)	19.5 (4.9–57.0)	0.977 (0.874–0.999)
Card Test (8%)	Complement Fixation (Cold)	0.917 (0.782–0.982)	0.937 (0.781–0.979)	0.952 (0.830–0.983)	0.890 (0.716–0.976)	19.8 (5.0–57.0)	0.984 (0.889–0.999)
Fluorescent Polarization Assay	Buffered Acidified Plate Antigen	0.556 (0.421–0.685)	0.829 (0.693–0.932)	0.818 (0.676–0.927)	0.573 (0.442–0.700)	4.5 (2.1–12.2)	0.731 (0.616–0.836)
Fluorescent Polarization Assay	Card Test (8%)	0.480 (0.347–0.617)	0.931 (0.828–0.987)	0.905 (0.771–0.982)	0.565 (0.444–0.684)	9.6 (3.3–53.0)	0.767 (0.663–0.854)
Fluorescent Polarization Assay	Complement Fixation (Cold)	0.564 (0.431–0.694)	0.753 (0.605–0.876)	0.759 (0.615–0.880)	0.556 (0.421–0.689)	3.2 (1.6–7.3)	0.685 (0.570–0.796)
Buffered Acidified Plate Antigen	Complement Fixation (Cold)	0.980 (0.910–0.984)	0.833 (0.635–0.970)	0.890 (0.752–0.981)	0.967 (0.855–0.977)	8.2 (3.0–49.0)	0.950 (0.848–0.999)
Card Test (8%)	Buffered Acidified Plate Antigen	0.898 (0.757–0.981)	0.936 (0.778–0.979)	0.952 (0.831–0.983)	0.869 (0.690–0.975)	19.8 (4.9–56.0)	0.987 (0.907–0.999)
Card Test (8%)	Complement Fixation (Cold)	0.918 (0.782–0.982)	0.936 (0.780–0.978)	0.952 (0.835–0.983)	0.890 (0.714–0.976)	20.0 (5.0–57.5)	0.950 (0.848–0.999)
Fluorescent Polarization Assay	Buffered Acidified Plate Antigen	0.556 (0.423–0.685)	0.831 (0.696–0.930)	0.817 (0.675–0.926)	0.573 (0.444–0.700)	4.5 (2.1–12.7)	0.931 (0.855–0.985)
Fluorescent Polarization Assay	Card Test (8%)	0.481 (0.347–0.616)	0.930 (0.828–0.987)	0.905 (0.772–0.982)	0.566 (0.444–0.685)	9.5 (3.3–55.0)	0.932 (0.854–0.985)
Fluorescent Polarization Assay	Complement Fixation (Cold)	0.564 (0.430–0.695)	0.753 (0.605–0.877)	0.759 (0.615–0.879)	0.556 (0.423–0.687)	3.2 (1.6–7.3)	0.926 (0.853–0.981)

Table S6. Diagnostic assay performance when two assays are interpreted in series.

Assay	Assay	Sensitivity	Specificity	Positive Predictive Value	Negative Predictive Value	Diagnostic Odds Ratio	Area Under Curve
Buffered Acidified Plate Antigen	Complement Fixation (Cold)	0.980 (0.914–0.984)	0.833 (0.635–0.969)	0.891 (0.754–0.981)	0.967 (0.857–0.977)	8.3 (3.1–50.0)	0.981 (0.884–0.999)
Card Test (8%)	Buffered Acidified Plate Antigen	0.898 (0.753–0.981)	0.936 (0.780–0.978)	0.952 (0.832–0.983)	0.869 (0.692–0.975)	19.5 (4.9–57.0)	0.977 (0.874–0.999)
Card Test (8%)	Complement Fixation (Cold)	0.917 (0.782–0.982)	0.937 (0.781–0.979)	0.952 (0.830–0.983)	0.890 (0.716–0.976)	19.8 (5.0–57.0)	0.984 (0.889–0.999)
Fluorescent Polarization Assay	Buffered Acidified Plate Antigen	0.556 (0.421–0.685)	0.829 (0.693–0.932)	0.818 (0.676–0.927)	0.573 (0.442–0.700)	4.5 (2.1–12.2)	0.731 (0.616–0.836)
Fluorescent Polarization Assay	Card Test (8%)	0.480 (0.347–0.617)	0.931 (0.828–0.987)	0.905 (0.771–0.982)	0.565 (0.444–0.684)	9.6 (3.3–53.0)	0.767 (0.663–0.854)
Fluorescent Polarization Assay	Complement Fixation (Cold)	0.564 (0.431–0.694)	0.753 (0.605–0.876)	0.759 (0.615–0.880)	0.556 (0.421–0.689)	3.2 (1.6–7.3)	0.685 (0.570–0.796)
Buffered Acidified Plate Antigen	Complement Fixation (Cold)	0.980 (0.910–0.984)	0.833 (0.635–0.970)	0.890 (0.752–0.981)	0.967 (0.855–0.977)	8.2 (3.0–49.0)	0.950 (0.848–0.999)
Card Test (8%)	Buffered Acidified Plate Antigen	0.898 (0.757–0.981)	0.936 (0.778–0.979)	0.952 (0.831–0.983)	0.869 (0.690–0.975)	19.8 (4.9–56.0)	0.987 (0.907–0.999)
Card Test (8%)	Complement Fixation (Cold)	0.918 (0.782–0.982)	0.936 (0.780–0.978)	0.952 (0.835–0.983)	0.890 (0.714–0.976)	20.0 (5.0–57.5)	0.950 (0.848–0.999)
Fluorescent Polarization Assay	Buffered Acidified Plate Antigen	0.556 (0.423–0.685)	0.831 (0.696–0.930)	0.817 (0.675–0.926)	0.573 (0.444–0.700)	4.5 (2.1–12.7)	0.931 (0.855–0.985)
Fluorescent Polarization Assay	Card Test (8%)	0.481 (0.347–0.616)	0.930 (0.828–0.987)	0.905 (0.772–0.982)	0.566 (0.444–0.685)	9.5 (3.3–55.0)	0.932 (0.854–0.985)
Fluorescent Polarization Assay	Complement Fixation (Cold)	0.564 (0.430–0.695)	0.753 (0.605–0.877)	0.759 (0.615–0.879)	0.556 (0.423–0.687)	3.2 (1.6–7.3)	0.926 (0.853–0.981)

Table S7. Logistic model performance and Bayesian *p*–values for predictors included in the models.

Assay	Bayesian R^2	LOO Adjusted R^2	Sex (Male)	Age (Subadult)	Strain (1330)
Buffered Acidified Plate Antigen	0.87	0.92	0.0665	0.2754	0.3431
Card Test (8%)	0.63	0.63	0.1897	0.0779	0.1586
Complement Fixation (Cold)	0.65	0.59	0.0398	0.0580	0.4530
Fluorescent Polarization Assay	0.87	0.88	0.0524	0.3367	0.3629

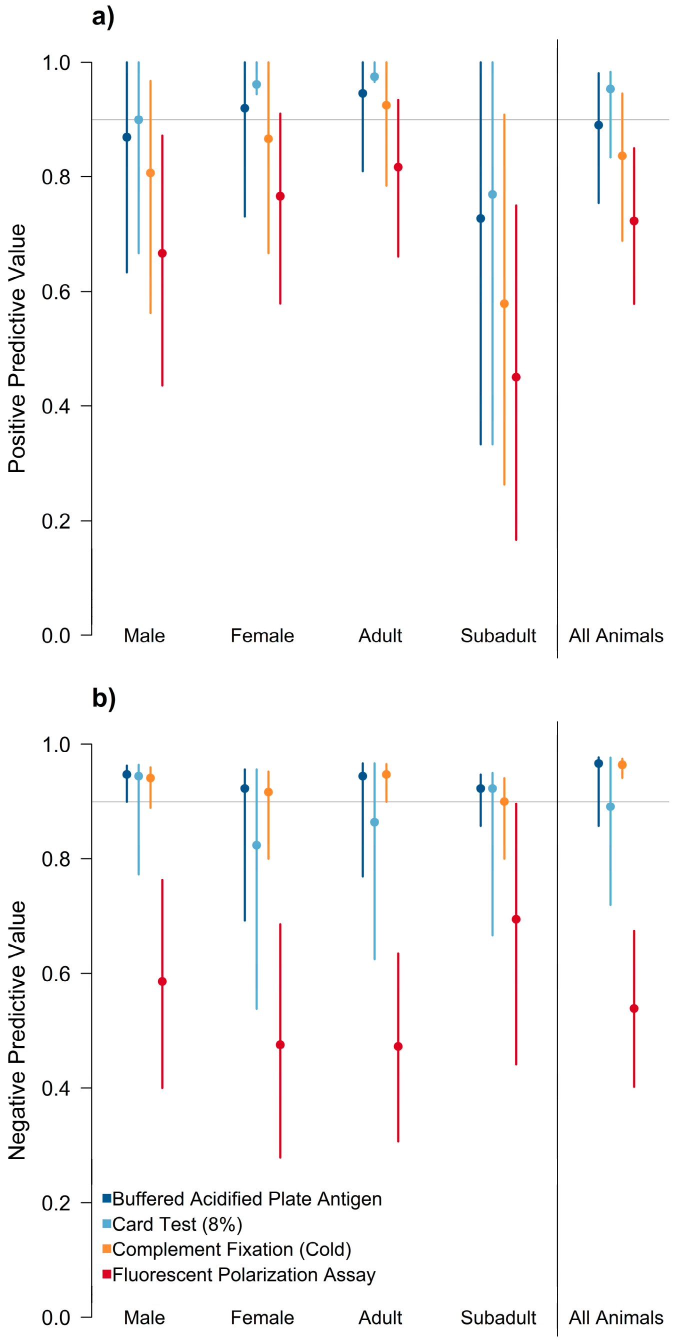

Figure S1. Positive and negative predictive values for the buffered acidified plate antigen test (BAPA), the 8% card agglutination test (card test), the fluorescence polarization assay (FPA) in tube format, and the *Brucella abortus/suis* complement fixation (CF) test. (**a**) Positive predictive value had generally large variation among age and sex except for the card test which had median above 0.95. All assays appeared to have the lowest positive predictive value for subadults although variance around the estimates was large. (**b**) Negative predictive value for three of the assays, BAPA, card

test, and CF, was generally above 0.90 with all having the highest negative predictive value for males.

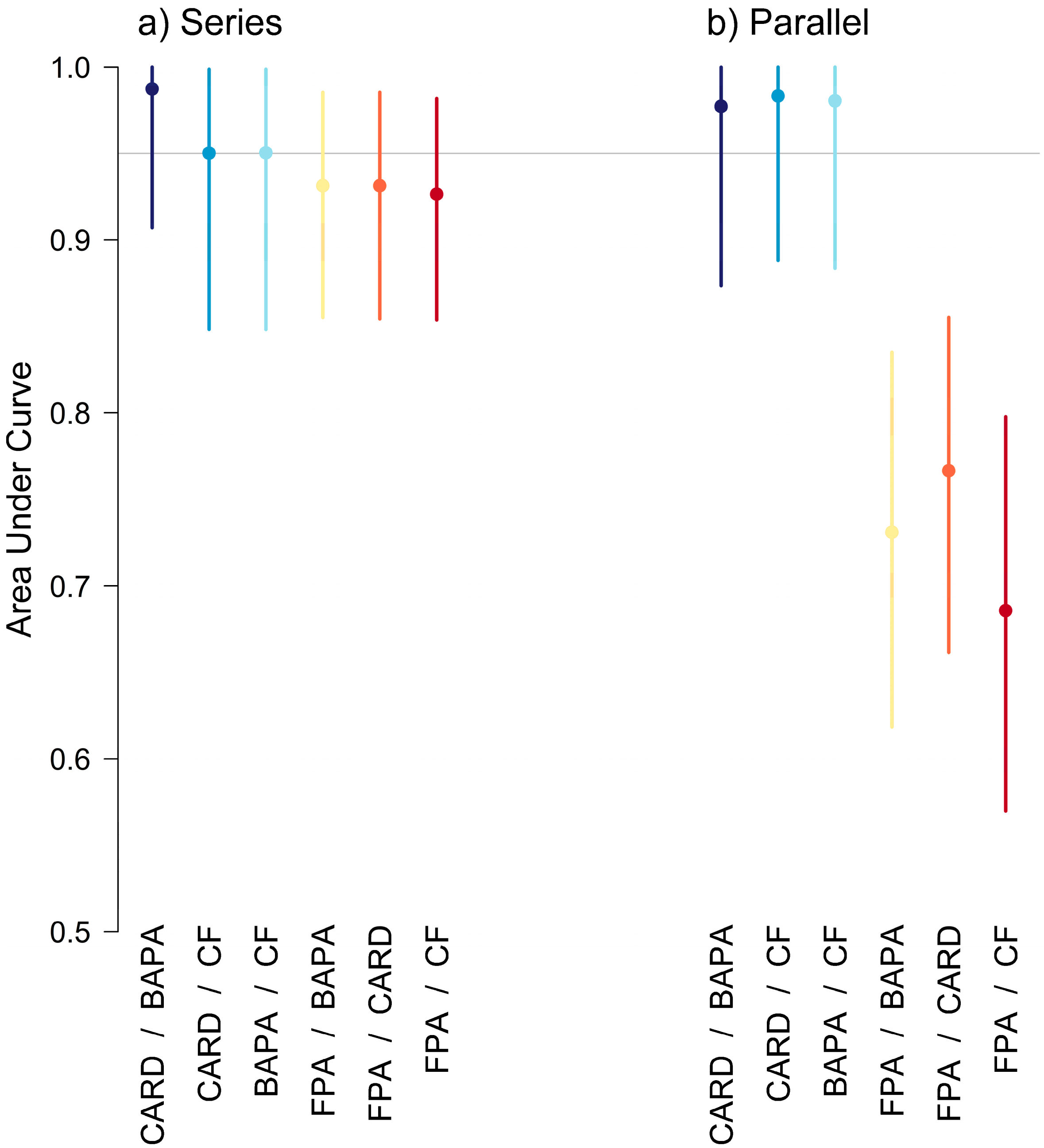

Figure S2. (**a**,**b**) The predictive capacity measured using area under the curve of the buffered acidified plate antigen test (BAPA), the 8% card agglutination test (card test), the fluorescence polarization assay (FPA) in tube format, and the *Brucella abortus/suis* complement fixation (CF) test when interpreted in parallel or series. The card test interpreted in series with BAPA had the highest area under the curve. However, the card test interpreted in parallel with BAPA or CF and BAPA interpreted in parallel also have high predictive capacity.

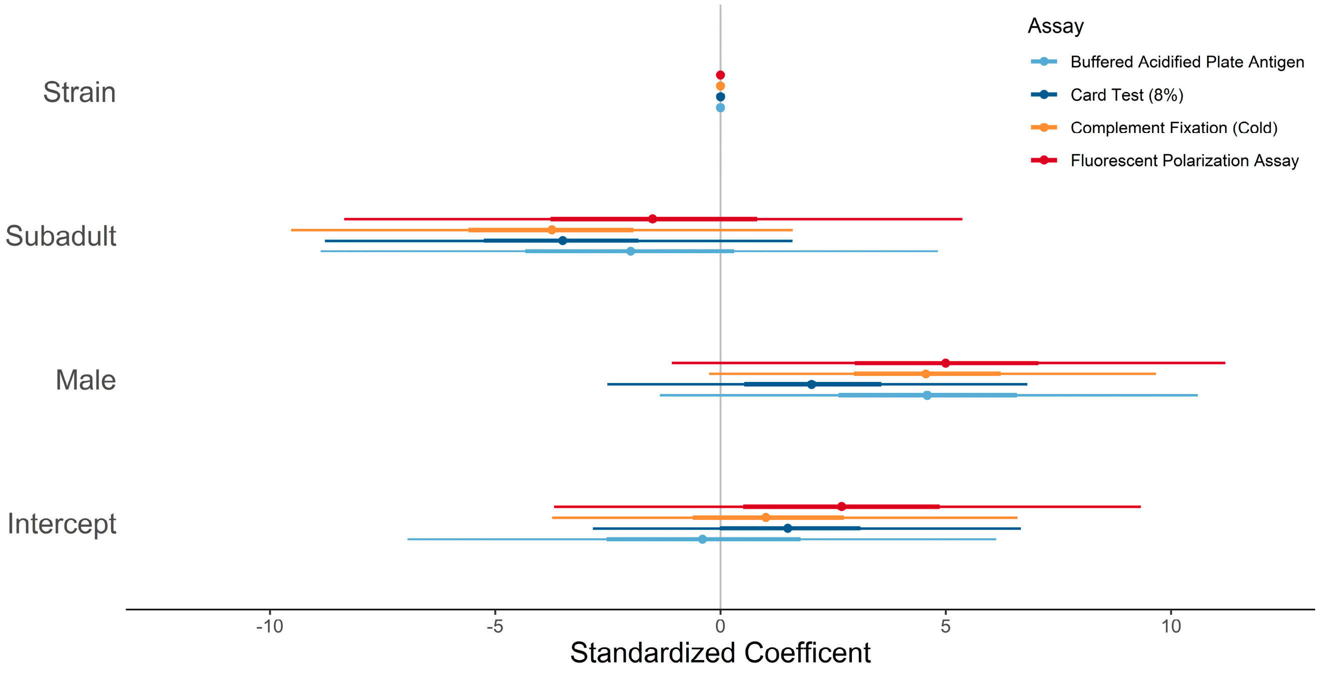

Figure S3. Predicted posterior distributions of standardized coefficients (median) with 50% credible interval (thick line) and 95% credible interval (thin line) for predictors used in the logistic models accounting for repeated measures. Coefficient values for subadult are relative to adult animals and coefficient values for male are relative to female animals.
